# Unlocking neuronal health: leveraging *C. elegans* for drug repurposing studies

**DOI:** 10.1186/s12964-025-02592-3

**Published:** 2026-01-15

**Authors:** Spoorthy Gowda, Arun Kumar, Ulrike Topf

**Affiliations:** 1 Laboratory of Molecular Basis of Aging and Rejuvenation, Institute of Biochemistry and Biophysics, Polish Academy of Sciences, Pawińskiego 5a, Warsaw, 02-106 Poland; 2 Doctoral School of Molecular Biology and Biological Chemistry, Institute of Biochemistry and Biophysics, Polish Academy of Sciences, Pawińskiego 5a, Warsaw, 02-106 Poland

**Keywords:** Healthspan, Nervous system, Drug repurposing, Anticonvulsant, Antidepressant, NSAID, *C. elegans*

## Abstract

Aging affects neuronal function, leading to both structural and functional changes in the human brain. The concomitant cognitive decline and neurodegenerative diseases drive efforts to find pharmaceutical drugs to ameliorate the consequences of neuron-associated deficits during aging. *C. elegans* is an excellent model to combine studies of the nervous system and drug screening. *C. elegans* has a well-characterized and relatively simple nervous system that allows for precise mapping of neuronal circuits. Neuronal activity plays a crucial role in regulating lifespan in *C. elegans*. Various genetic, environmental, and pharmacological factors modulate neuronal signaling, affecting longevity by activating cell non-autonomous mechanisms in other tissues. Drug repurposing through screening approved human drugs in model organisms is an increasingly applied approach to identify off-label therapeutic effects. This review discusses research in *C. elegans* that applied drugs as neuromodulators with the aim to increase lifespan and healthspan. We focused on drugs with confirmed anticonvulsant, antidepressant, antipsychotic, antihypertensive, or anti-inflammatory properties. This review highlights potential mechanisms of how such drugs can exert a beneficial effect during organismal aging. Future research must intensify efforts to not only demonstrate a beneficial effect of repurposed drugs, but more importantly, to elucidate the underlying cellular responses. This will be essential for advancing selected drugs that have the potential for conserved benefit in humans.

## Introduction

 The nematode *Caenorhabditis elegans* is an excellent model to study aging in a multicellular organism. Aging research uses two key terms, lifespan and healthspan, to quantify aging. Lifespan is the total duration of time an organism remains alive, from birth to death. Unlike lifespan, healthspan focuses on the quality of life and the duration of optimal physical and cognitive function. Genetic mutants with almost twice the lifespan of wild-type worms were discovered forty years ago, providing the initial spark for longevity research [1]. Since then, *C. elegans* development has been studied throughout its life cycle to understand the behavioral, morphological and molecular changes associated with the progression of age [2–4]. Aging is a process characterized by phenotypic changes observed in the organism over its lifetime [5, 6]. Recently, aging studies have refocused on phenotypes and mechanisms at the tissue level in order to understand tissue-specific declines and cross-talk between tissues that may affect the quality of health of the whole organism [7, 8]. *C. elegans* phenocopies human aging symptoms like loss of cognitive functions including memory and sensory functions and eventually loss of motor functions late in life [reviewed in [9]].

The reversal or halting of the aging process in living organisms to restore a youthful state is referred to as rejuvenation [10, 11]. A rejuvenation strategy depends on targeting multiple cellular processes. Testing approved drugs and medications has accelerated the rejuvenation avenue [12, 13]. Hereby, drugs are repurposed for uses beyond their labelled medical scope [14]. Known pharmaceutical drugs target specific molecules or tissues, but the broad effects of many drugs on the molecular levels are often not fully understood [15]. Moreover, combination of different drugs and a shift in focus towards treatment of aged organisms rather than tests on young animals has become a newly emerging field in biology [13, 16, 17]. Tissue-level improvements with genetic mutants and pharmaceutical drugs are gaining focus for its targeted approach to rejuvenation [4, 18, 19].

In *C. elegans*, neurons receive and process sensory information to navigate through its environment to find and distinguish between different foods and stay away from predators. Given that neurons integrate and propagate environmental and internal cues to the whole body, it is a powerful starting point to impact health at the organismal level. The body movement of *C. elegans* has been used as a direct parameter of health and an indirect parameter of lifespan and longevity [20]. Combining behavioural data, which are dependent on movement, with neuron-centric approach has provided a nervous system-muscle crosstalk for understanding health and lifespan [21]. These two tissues are important for humans to maintain a good quality of life. As a predominantly post-mitotic tissue, neurons are sensitive to protein homeostatic changes and communicate dysfunctions to non-neuronal tissues [22]. Thus, utilising drugs that can modulate neuronal health might have an impact on other tissues. Given that *C. elegans* has been one of the organisms with a fully mapped nervous system, it has become an ideal model for drug testing, particularly to combat neuronal aging [8, 19, 23, 24]. *C. elegans* has been used to model many human age-linked neurodegenerative diseases like Alzheimer’s and Parkinson’s diseases, thus providing a basis in worms to investigate human age-related neurological changes [25–27]. These models allow investigation of conserved pathways of neurodegeneration and therapeutic responses in vivo. This review focuses on nervous system aging and summarizes research using *C. elegans* that have tested approved drugs for humans for their potential to rejuvenate the nervous system health.

## Characteristics of neuronal aging in *C. elegans*

### Morphological and functional changes

**Fig. 1:**
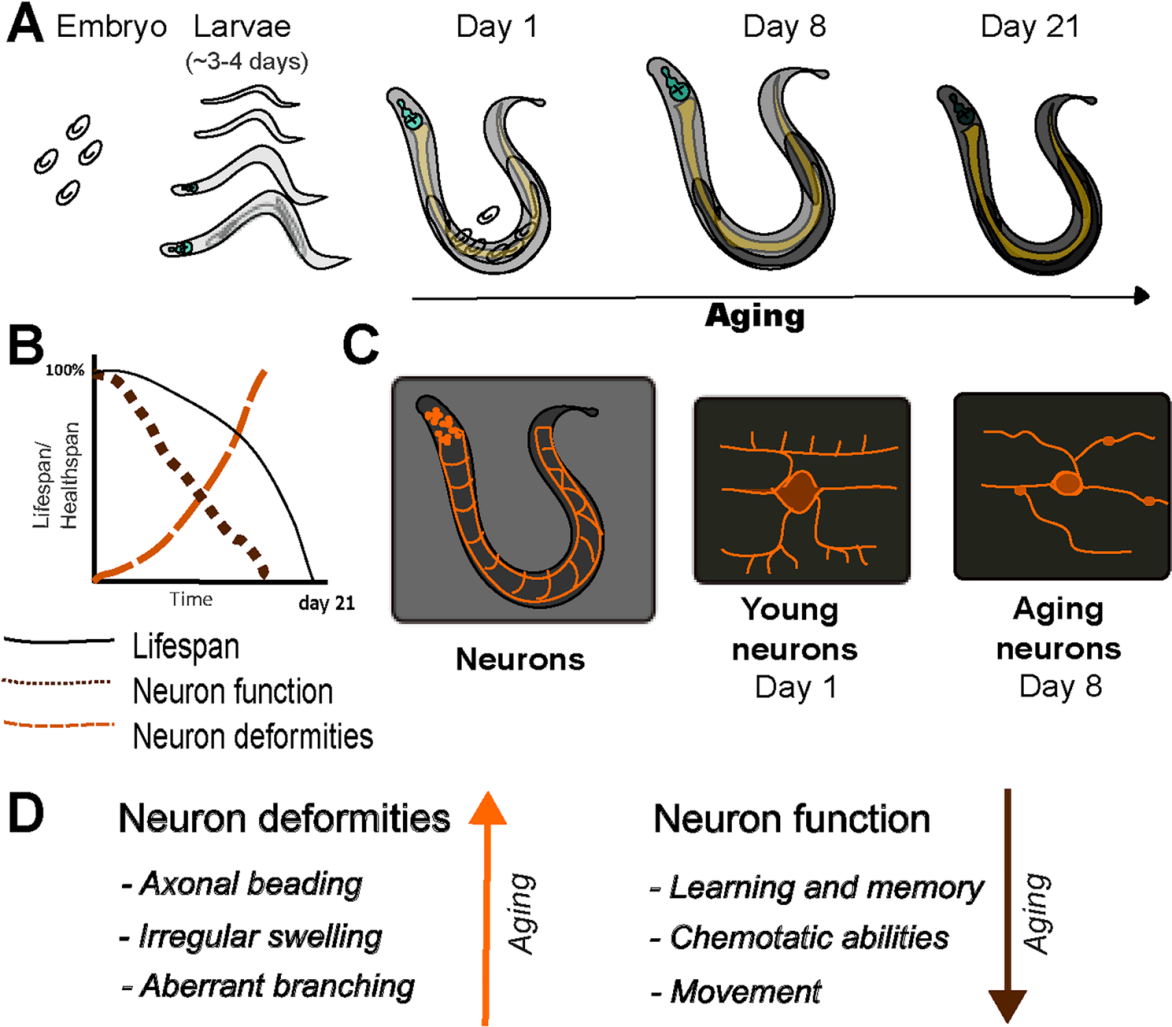
*C. elegans* aging at the morphological and molecular scale.** A** *C. elegans* begins life in the embryonic stage and progresses through four larval stages before reaching adulthood. The average lifespan of adult worms is 21 days at 20 °C, represented in the graph by the black line. **B** Functional decline and morphological deformities are depicted on the same graph as the lifespan to show the progression of these events. **C** A schematic representation of *C. elegans* neurons is shown in orange. The neurons depict PVD motor neurons on days 1 and 8 of adulthood [37]. **D** Deformation of neurons increases whereas neuronal function decreases during aging (indicated by arrows) [reviewed in [9]. For visual representation of neuronal deformities, refer to the following publications [38–41]

The nervous system of *C. elegans* consists of 302 neurons in hermaphrodites and 385 neurons in males [28, 29]. The development of the nervous system takes place during the early stages of the worm life cycle, which consists of embryo, four larval stages and an adult [30–32]. Worms survive for approximately 21 days after reaching adulthood when animals are grown at 20 °C under laboratory conditions (Fig. 1A) [7]. The *C. elegans* nervous system has been mapped entirely, including neuronal connections. The functions of individual neurons have been well characterized at the cellular and molecular level [23, 33]. *C. elegans* neurons are categorized according to their functions including sensory neurons, motor neurons, interneurons and polymodal neurons [34]. Sensory neurons are further divided into eight categories: chemosensors, olfactory sensors, oxygen sensors, nociceptors (pain receptors), thermosensors, mechanosensors, proprioceptors and neurons of unknown modality [28, 35, 36].

Neuronal age-based decline is characterized by morphological and functional deterioration (Fig. 1B). Morphologically, aging neurons exhibit increased cellular deformities such as reduced synaptic vesicles, blebbing and beading of axons and defective or extra-neuronal processes in various neuronal groups (Fig. 1C) [reviewed in [9, 42]. Morphological changes observed in day 8-old adults compared to day 1-old adults leads to decreased functionality of neurons. Functional decline includes decrease of olfactory-based learning and memory, isothermal tracking and chemosensation [reviewed in [9] (Fig. 1D).

Studying neuronal morphology during aging has been facilitated by advances in electron microscopy and the use of fluorescent reporter strains [27, 43, 44]. Continually evolving visualization techniques help to understand morphological changes, but most importantly allow the quantification of neuronal activity. Beyond morphological assessment, neuronal activity has been visualized by calcium imaging techniques using the fluorescent calcium sensor GCaMP expressed in neurons [45]. This sensor has helped to visualize the excitatory and inhibitory states of all neurons in the worm. With increasing age of the worm, the sensor showed a shift towards excitatory activity in the neurons (especially in the head region), which shifted the excitation-inhibition balance. Aging neurons also showed an increased period of quiescence compared to young adults (day 9 vs. day 1 of adulthood) [46]. These observations indicated neuronal dysregulation in the worm as it ages. This excitatory-inhibitory imbalance is likely linked to dysregulation in synaptic transmission and altered calcium homeostasis, which may underlie observed impairments in behavior and sensory function.

Interestingly, genetic mutants impairing neuronal function or removal of neurons prolonged lifespan [35, 36, 47, 48]. While inactivation of neurons promotes longer life, important questions remain about its benefit for overall healthspan.

### Molecular changes

The aging process causes significant molecular changes at the transcript and protein levels. These molecular changes occurred during the different life stages of worms from young (fourth larval stage – L4), middle (day 6 of adulthood) to old age (day 15 of adulthood) and modulated important signalling pathways, neuronal function and proteostasis. During the course of aging, the expression of genes within several signalling pathways, such as MAPK, Wnt, mTOR and TGF-β pathways were downregulated, especially in the middle and older-aged worms compared to young worms. These pathways regulate development, metabolism and cell-type specific functions [49]. Development of single-cell RNA sequencing techniques allowed characterization of transcriptomic changes specific in neurons. Age-related changes in neuronal morphology and function are accompanied by remodeling of gene expression during the aging process (day 1 vs. day 8 of adulthood). Genes linked to proteasome proteolysis mechanism such as E3 ligases, the response to oxidative stress and transcription factors are upregulated during aging [50]. This upregulation may reflect a compensatory stress response to accumulating misfolded or damaged proteins. However, over time, the proteostasis network becomes insufficient to manage cellular burden, leading to dysfunction. Conversely, the genes encoding extracellular proteins, involved in neuronal signalling, transmembrane transport and cytoskeleton are downregulated [51, 52]. This may contribute to the loss of signal fidelity and structural integrity in aging neurons. Moreover, the unfolded protein response of the endoplasmic reticulum (UPR^ER^) was downregulated in the neurons of older worms [53]. This downregulation compromises the ability of neurons to restore proteostasis under the stress condition, which may stimulate neurodegeneration.

Functionally, *C. elegans* showed a decrease in short-term associated memory on day 7 compared to day 1 of adulthood [54]. Sex-based differences showed that males had a relatively faster decline in neuronal function, evident by changes in neuronal morphology and chemotaxis behavior [55]. Transcriptomic analysis showed that aging hermaphrodites (day 7 vs. day 1 of adulthood) showed upregulation of genes involved in proteasome-related components, stress defense and histones [54, 55]. In aging males (day 8 vs. day 2 of adulthood), transcripts linked to the GPCR receptors, stress response, ribosome biogenesis and proteasome activity were upregulated [55]. Sex-specific trajectories in gene expression and function may stem from differences in reproductive strategies, metabolic demand, and hormonal regulation, warranting further investigation into sex as a biological variable in neuro-aging.

Comprehensive proteomics analyses have complemented and extended the observed transcriptomic changes during *C. elegans* aging. Aging causes dysregulation in protein homeostasis (proteostasis) affecting protein synthesis, degradation and folding [56–58]. Dysregulation of the proteostasis network affects neurons by increasing damage to cellular components, which ultimately negatively affects the organism’s health. Translation became dysregulated and ribosome pausing on mRNA increased (day 12 vs. day 1 of adulthood) [59]. Ribosomal protein turnover increased within the first week of adulthood. Ubiquitin-proteasome dependent degradation of proteins decreased as worms aged (day 15 vs. day 1 of adulthood) [60] despite that proteins involved in the ubiquitin-proteasome system remained stable during early aging. This suggests that the degradation system becomes overwhelmed when proteins form aggregates [61]. The decline in the unfolded protein response (UPR) with age exacerbates the problem [62]. The turnover of molecular chaperones and oxidative stress defense proteins remained stable during the first week of adulthood [61]. Neuron specific proteomics was achieved by utilising proximity labelling methods in *C. elegans* [63, 64]. An improved version of *E. coli* biotin ligase (TurboID) fused to cellular proteins, allowing pan-neuronal, neuron-specific or compartmental expression provided new insight into the neuronal aging proteome [65]. The results indicated increased vesicle turnover in response to age-related damage or neuronal stress. Proteins related to neurotransmitter transport were upregulated in day 12 vs. day 1 adult worms. This may suggest that compensatory mechanisms are activated for maintaining neurotransmission in aging neurons. Downregulated proteins in day 9 and day 12 vs. day 1 included ribosomes and other proteins taking part in translation, similar to trends found in whole worms. This likely shows diminished ability to synthesize proteins required for synaptic maintenance and repair. Morphological and molecular changes occur in parallel leading to the aging phenotype in neurons and hence, the whole organism [37]. Integrating proteomic signatures with behavioral and functional phenotypes may help to map causal links between protein expression and neural decline.

The breakdown of proteostasis can lead to neurodegenerative diseases in humans. Diseases such as Parkinson’s, Alzheimer’s, frontotemporal dementia and Machado-Joseph (MDJ) neurodegeneration have been modelled in *C. elegans* [reviewed in [38, 66]. Loss of protein homeostasis has been monitored in *C. elegans* by expressing aggregation-prone proteins including human proteins linked to neurodegeneration. Such proteins have often been expressed in the muscles to facilitate the generation of movement defects. The transgenic models have shown progressive paralysis with increasing age and protein aggregation and thus, have been useful to screen for therapeutic interventions [25, 26].

## Organismal rejuvenation through modulation of neuronal activity

**Fig. 2:**
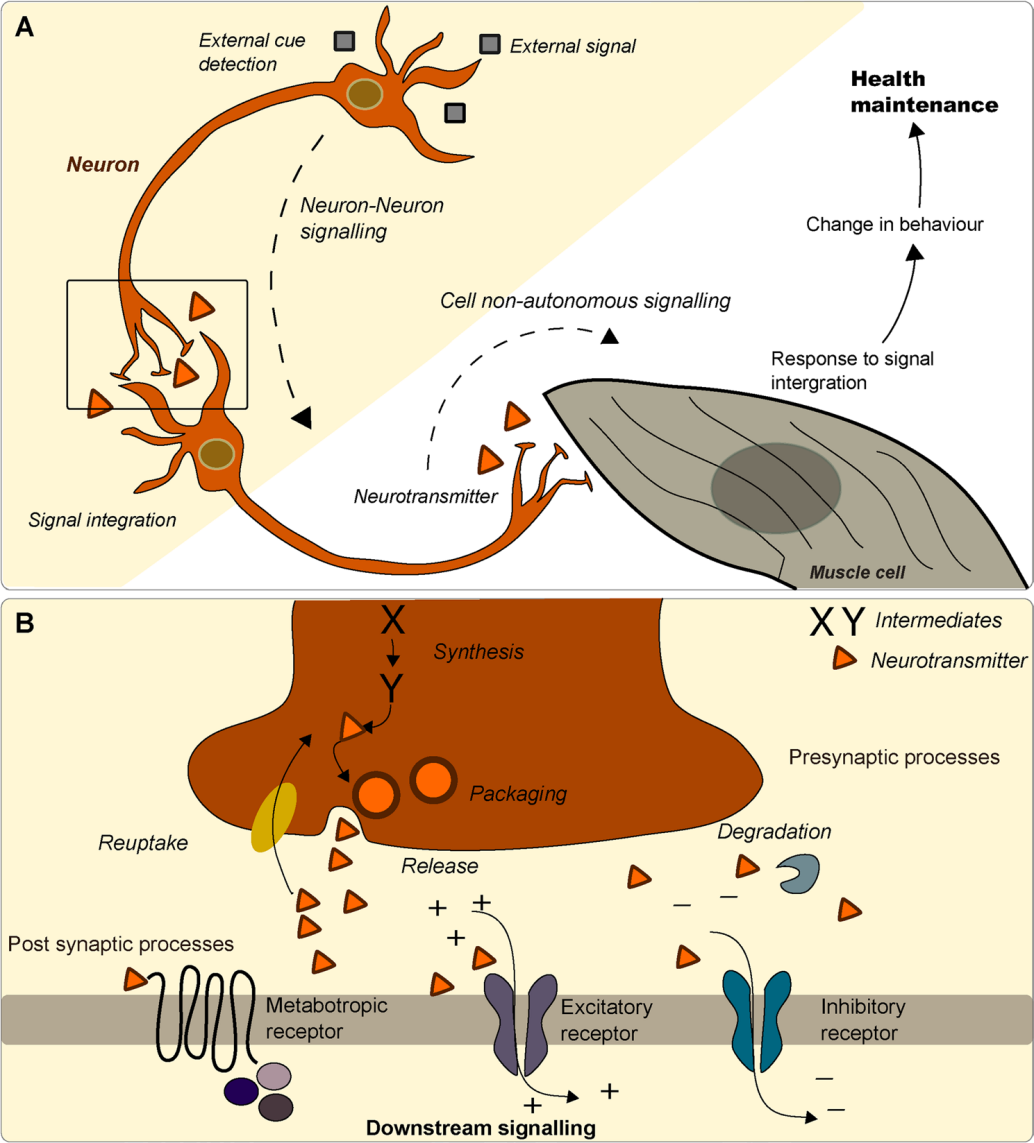
Neuron function and inter-tissue communication. **A** Neuron detects external signal and transduces the signal to other neurons or other tissues such as muscles. The yellow background indicates neuron-neuron signalling and white background indicates cell non-autonomous signalling. **B** Enlarged inset of panel A depicting signalling at the synapse. The synapse shows the synthesis of presynaptic neurotransmitters (via intermediates X and Y), as well as their packaging, release, and reuptake. These are all presynaptic processes. The receptor binding and signal transduction is a postsynaptic process. Metabotropic (GPCR) and ionic receptors relay neurotransmitter signal to the postsynaptic cell. Degradation of the neurotransmitter occurs at the synaptic cleft. The ions, cations [+] and anions [–] signs denote $\:{\mathrm{N}\mathrm{a}}^{+},\:{\mathrm{K}}^{+},\:{\mathrm{C}\mathrm{a}}^{2+}$and $\:{\mathrm{C}\mathrm{l}}^{-}$, respectively

Tissue-specific decline during aging affects the health and fitness of other tissues through inter-tissue signalling mechanisms [reviewed in [67]. Neurons integrate and transmit various exogenous and endogenous signals to carry out specific functions such as sensory cue detection, signal integration, and movement (Fig. 2A). In order to transmit signals the presynaptic, post-synaptic and intercellular components of neurons have to work together. Presynaptic functions include neurotransmitter packaging, release, reuptake, and degradation. At the post-synaptic compartment, neurotransmitter signals are transduced by ligand-gated ion channels (ionotropic receptors) and receptors that require G proteins and second messengers to indirectly modulate ionic activity in neurons (metabotropic receptors) (Fig. 2B). Intracellular components regulate neurotransmitter metabolism and signal transduction across the axon [33, 68]. Neuromodulation is a physiological process by which a given neuron uses one or more chemicals to regulate diverse population of neurons. The neuromodulators can alter intrinsic firing patterns, voltage-channel dependent currents, synaptic efficacy and synaptic connectivity [69]. Neuromodulators are a subset of neurotransmitters that act in a systemic manner and their action has long-lasting effects. *C. elegans* expresses all major classes of neuromodulators, including biogenic amines (dopamine, serotonin, octopamine and tyramine), acetylcholine, neuropeptides and cytokines (such as TGF-β and the interleukin IL-17). Neuromodulators are released from the pre-synaptic site and bind to receptors on the post-synaptic site to exert health and longevity. Early studies in *C. elegans* have revealed that alterations in the nervous system are beneficial for longevity [35, 70–73].

Neurons communicating with non-neuronal tissue has been a part of cell non-autonomous signalling [reviewed in [67] (Fig. 2A). Recent studies have highlighted the complex role of cell non-autonomous mechanisms between neuron-tissue communication networks, which may play an important role in organismal aging. Several key mechanisms by which neurons communicate with non-neuronal tissues include peripheral insulin/IGF-1 pathway modulated by insulin-like peptides [74], lipid metabolism [75], mitochondrial function regulated by serotonergic neurons [76], long-range signalling through metabolite transporters [77] and extracellular vesicle-based communication [reviewed in [78]. In the past decade, researchers have focused on the role of protein homeostasis as an important mechanism by which neuronal signalling can affect the systemic aging in non-neuronal tissues of organisms. This has been characterised by high protein aggregation and reduced cellular function during age-associated decline, leading to increased susceptibility to proteotoxic stress [79]. Neuronal signalling may improve resistance to proteotoxicity in distant non-neuronal tissues. Beneficial responses are mediated through activation of stress responses such as UPR modulation in both mitochondria and endoplasmic reticulum [80, 81], or activation of the heat shock response by HSF-1, which improved chaperone-based protein folding in intestine and muscles [82, 83]. Other peripheral tissue responses have been mediated through exosomes carrying microRNA and improved heat shock response in distant tissues [84]. Exosomal communications have also been shown to regulate circadian rhythm leading to regulation of systemic proteostasis [85].

Recent studies have demonstrated the role of various neurotransmitters in maintaining tissue health. Serotonergic pathways have improved mitochondrial function and autophagic flux [86, 87]. Dopaminergic signalling has played a crucial role in modulating cellular stress responses and has regulated proteolytic degradation [88, 89]. GABAergic signalling has contributed to proteostasis maintenance [90] and cholinergic signalling has been able to regulate systemic stress responses [91]. Moreover, new variants of serotonin receptors that have primarily mediated signalling processes related to aging were identified [92]. A detailed understanding of systemic aging and neural signalling can help to reveal how neural activity patterns effect the organismal longevity and tissue health, thus providing opportunities for treating age-associated diseases. Therefore, this review will focus on neuronal modulation aimed at restoring the balance between neural excitation and inhibition, and its impact on protein homeostasis in tissues through pharmacological means.

## Pharmacological approaches to neuromodulation

To restore the health of aging tissues and organisms to a youthful state, pharmacological neuromodulation represents a promising rejuvenation strategy. Drugs treating a disease state (e.g. depression, epilepsy, psychosis) often act by targeting neurotransmitter systems. Many of these drugs have also promoted longevity and healthspan in *C. elegans*, thus providing a potential platform for drug repurposing. Drug repurposing has been used to accelerate drug research and approval time [14]. Several studies have showed the use of *C. elegans* in screening for pro-longevity drugs [14, 16, 19, 93–97].

**Fig. 3:**
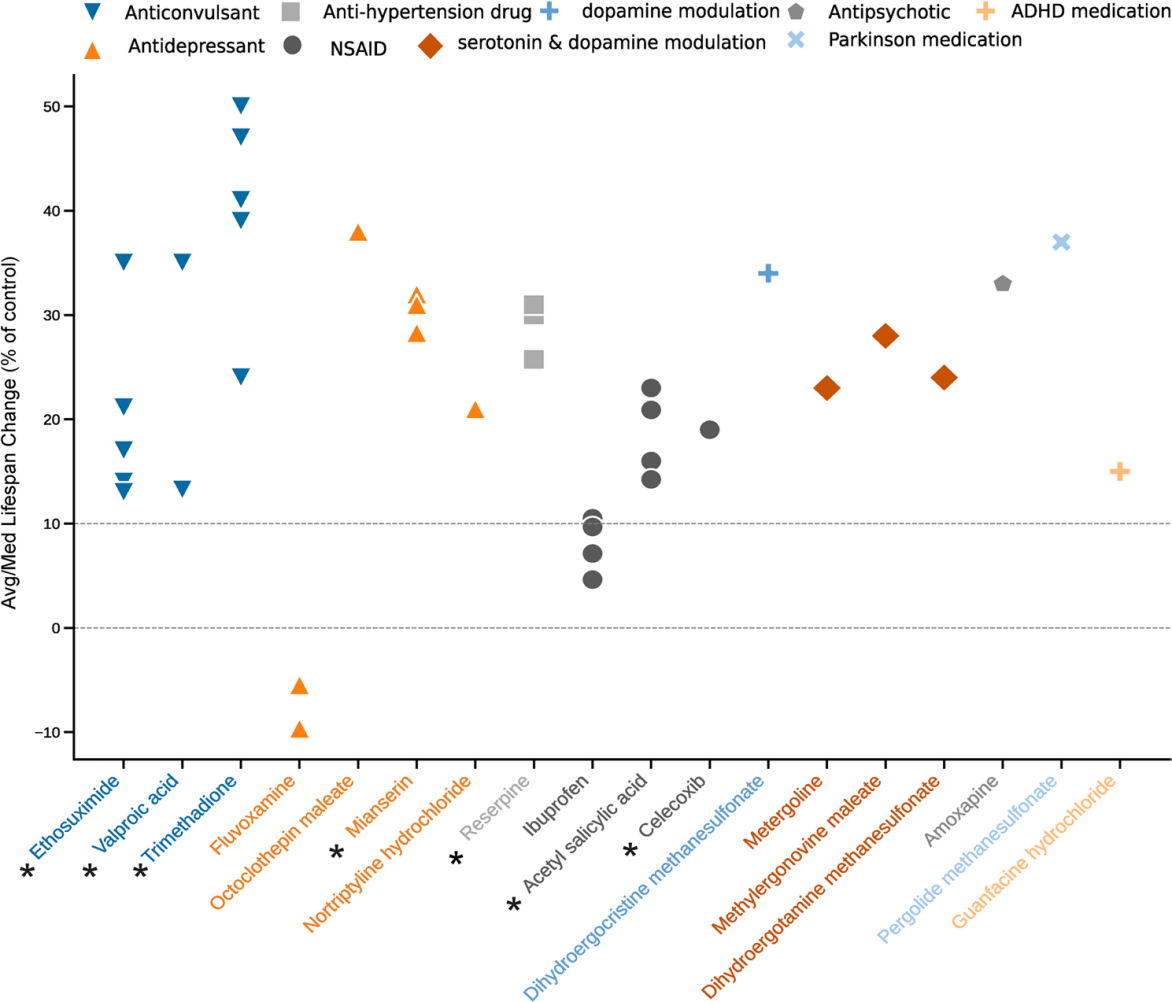
Lifespan change in *C. elegans* upon drug treatment based on data in DrugAge database. The average or median (Avg/Med) lifespan values were filtered for statistical significance from the DrugAge database. Each data point represents the result of one assay. Experiments may vary by dosage and strain. The dotted line represents a 10% cut-off for drug selection. The categories are based on drug descriptions in PubChem. The main text of the review evaluates drugs marked with an asterisk due to the larger number of available research on their effects on *C. elegans*

Pharmacological agents that target neuromodulation are considered for this review as they manipulate neural activity. Given that dopamine, serotonin and acetylcholine neuromodulators are some of the most targeted in the human nervous system to combat disease, we have selected a group of neuromodulating drugs based on their reported lifespan-extending effects in *C. elegans*, sourced from DrugAge database ([98], https://genomics.senescence.info/drugs/) (Fig. 3). Only drugs demonstrating ≥ 10% lifespan-extending effects with statistical significance were retained and their detailed mechanisms are discussed for a subset of drugs with substantial available literature. We took a special consideration for NSAIDs, due to the role of neuroinflammation as a precursor to neurodegeneration and inflammation as one of the hallmarks of aging [reviewed in [99–101].

Neurological medications have been prescribed for specific human diseases and include among others, anticonvulsant, antidepressant, and antipsychotic drugs. Other classes of drugs used to treat ailments in humans are antihypertensive and non-steroidal anti-inflammatory drugs (NSAID). According to the medical databases NIH Cancer (www.cancer.gov) and PubChem (https://pubchem.ncbi.nlm.nih.gov/), these drug classes have had the following basic definitions:

I.  *Anticonvulsant –* a drug that modulates electrical activity of the brain during convulsions or seizures. Convulsions are periods of rapid contractions and relaxations of the muscles, which lead to uncontrolled shaking of the body.
II.  *Antidepressant –* a drug used to treat depression; depression can be described as a medical state of ongoing feelings of despair, sadness, loss of energy and difficulty in maintaining daily life activities.
III. *Antipsychotic drug –* a drug used to treat symptoms of psychosis by blocking specific chemicals in the nervous system. Basic symptoms of psychosis include hallucinations (seeing or hearing things that aren’t there), dementia (loss of memory) and delusions (false beliefs).
IV. *Antihypertensive drug –* a drug used to reduce high blood pressure; many drugs use different mechanisms of action to reduce the blood pressure of the individual.
V. *NSAID (Non-Steroidal Anti-Inflammatory Drug) –* a drug used to reduce swelling, pain and fever not dependent on steroid-based mechanisms.

Interestingly, examples of approved medication and drugs within the classes described above have been tested in *C. elegans* models of neurodegeneration and have shown neuroprotective effects [102–105]. In the following sections, we outlined fundamental findings of the effect of selected drugs that have significant research progress on *C. elegans* physiology and evaluate the drug’s potential to restore features of youth in aged worms.

## Anticonvulsant

### Ethosuximide – a succinimide based anticonvulsant

Ethosuximide (ETX) is a succinimide anticonvulsant that has been shown to improve lifespan at doses of 4 mg/ml and 2 mg/ml when administered from the last larval developmental stage (L4) until death (Fig. 3) [106]. Along the increase in lifespan, healthspan parameters related to neuromuscular function showed improvement including increased body movement and pharyngeal pumping rate. ETX increased the frequency of egg laying at an earlier stage (1-cell to 7-cell stages) compared to untreated wild-type worms that generally lay eggs at the 30-cell stage of development. These physiological changes indicate enhanced muscle function stimulated by the neuronal network. Indeed, inhibiting the function of the HSN neuron that innervates the vulva muscles and is necessary for egg laying, abrogated the early-stage egg laying upon ETX treatment [106]. This provided proof that ETX acted presynaptically. ETX treatment reduced chemo-sensation in wild-type worms (Table 1). These two assays highlight the neuromodulation capacity of the drug beyond humans. Furthermore, an unbiased screen revealed that chemosensory neuron function mediated by the genes *osm-3* and *che-3* was required to survive a lethal dose of ETX. In addition, ETX treatment inhibited the formation of the stress-resistant dauer developmental stage induced in the animals by sensing a high-density starved population of worms, a response that was also observed in *osm-3* mutants [107]. Decreased chemo-sensation was shown to extend lifespan through inhibition of insulin/IGF-1 signalling and consequent increase in DAF-16/FOXO function [35, 108]. DAF-16 is the sole ortholog of FOXO family transcription factors and activates genes involved in longevity, lipogenesis, heat shock survival and oxidative stress response [reviewed in [109]. However, ETX-depended lifespan extension in wild-type worms was partially independent of DAF-16 function, suggesting additional pathways may contribute to its lifespan-extension. While ETX failed to extend lifespan of *osm-3* mutants, consistent with data from chemo-sensation assays, ETX-depended lifespan extension was only partially depended on genes important for neurotransmission (*unc-31*, *unc-64* and *aex-3*) [106, 107]. This indicates that ETX acts through multiple molecular targets beyond common neurotransmitter pathways. Identification of the target (or targets) of ETX should help to elucidate the mechanism in worms.

**Table 1:**
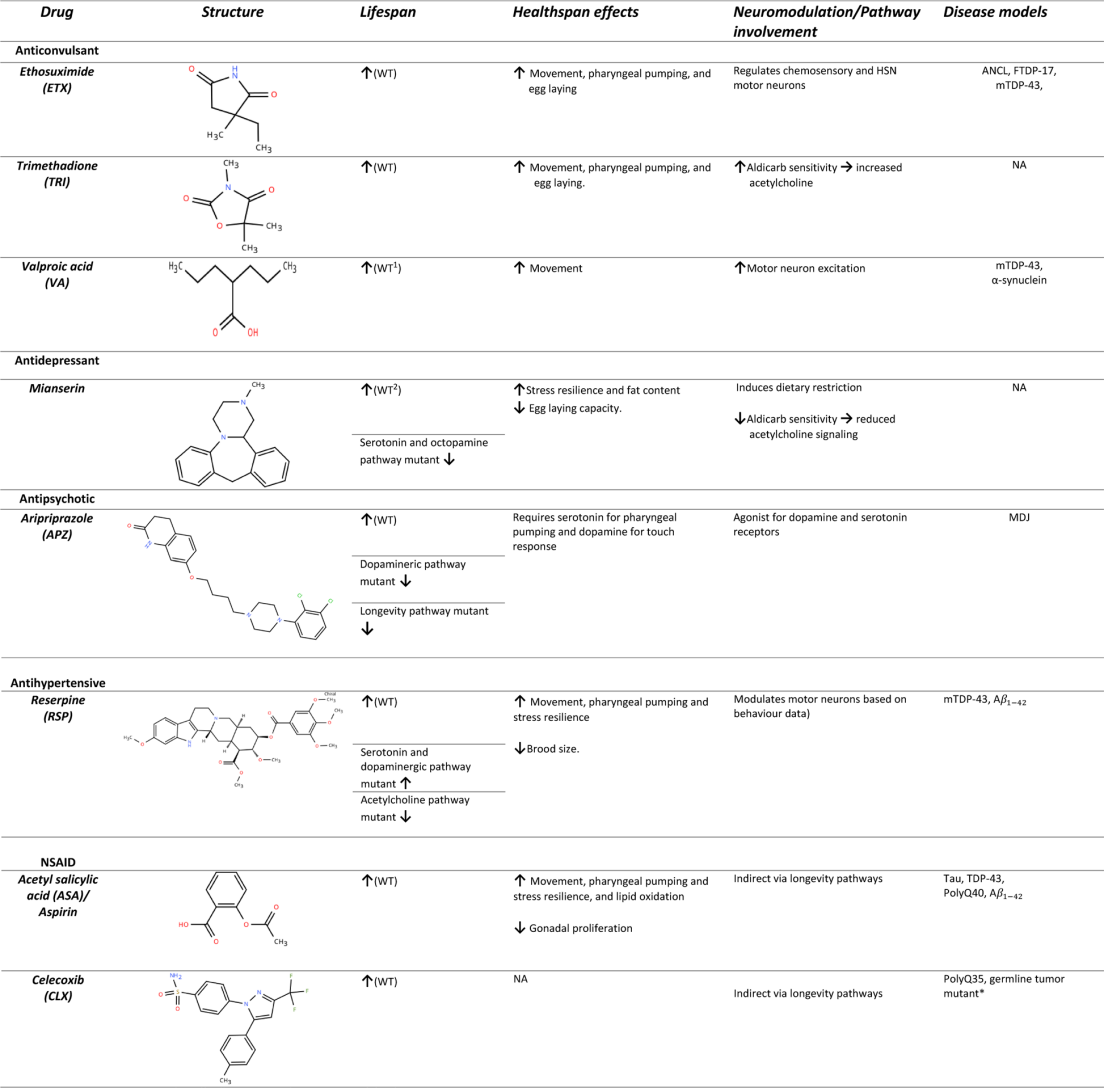
Neuromodulatory drugs tested in *C. elegans*. Chemical structures were obtained from chemspider (https://www.chemspider.com/StructureSearch*).* Lifespan effects are indicated by arrows: an upward arrow ($\uparrow$) represents an increase in lifespan, while a downward arrow ($\downarrow$) indicates a decrease. A reduction in lifespan associated with specific pathways denotes the dependency of the pathway for the drug’s health-promoting effects. The healthspan column reports behavioural and physiological changes commonly assessed in *C. elegans* drug studies. The “Disease” column lists the neurodegenerative protein models used for drug testing, with a germline tumor model included only for celecoxib. ^1^Effective when worms were fed with dead bacteria. ^2^Effective during reproductive period. Hermaphrodite specific motor neuron (HSN); ANCL – Autosomal-dominant adult-onset neuronal ceroid lipofuscinosis; FTDP-17- frontotemporal dementia with parkinsonism-17; mTDP-43- amyotrophic sclerosis model with mutant TAR DNA-binding protein-43; MDJ - Machado-Joseph neurodegenerative disease; amyloid beta protein - A$\:{{\upbeta\:}}_{1-42}$. WT – Wild type (N2); NA – no available data. For references, refer to Sections "Anticonvulsant", "Antidepressant", "Antipsychotic drug", "Antihypertensive drug", "NSAID"

The suggested target of ETX in mammalian system is a T-type calcium channel [110, 111]. The predicted *C. elegans* homolog is the *cca-1* gene encoding for a low voltage-T-type gated calcium channel expressed in alimentary muscles, distal tip cells, pharyngeal and serotonergic neurons and ventral nerve cord. However, a loss-of-function mutant of *cca-1* failed to inhibit the ETX-depended lifespan extension or suppressed early egg-laying phenotype [107, 112]. Thus, further research in *C. elegans* might reveal other direct targets of ETX that will facilitate discovery of mechanisms underlying the healthspan benefits.

Importantly, ETX treatment improved both lifespan and healthspan in multiple *C. elegans* models of neurodegeneration and demonstrated translational potential of this invertebrate model [103, 113, 114] (Table 1). Autosomal-dominant adult-onset neuronal ceroid lipofuscinosis (ANCL) is modelled in *C. elegans* by mutating the worm homolog of DNAJC5 (*dnj-14*), a neuronal chaperone of the DnaJ/Hsp40 family. ETX-dependent lifespan extension in *dnj-14* mutants required *daf-16* function similar to observations in wild-type worms [103]. ETX showed improvement in chemotaxis of both homozygous and heterozygous *dnj-14* mutants [114]. Microarray data in *dnj-14* mutant after long-term exposure to ETX at 1 mg/ml revealed upregulation of downstream targets of *daf-16*. The dependence on *daf-16* was shown via RNA interference in the disease model with weak nuclear localisation of the DAF-16 protein indicating transcriptional activation. Interestingly, the ability of ETX to promote DAF-16/FOXO target gene expression was confirmed in mouse neuroblastoma cell line upon treatment with ETX (0.5 −1 mg/ml), suggesting conservation of mechanisms across species and supporting translational applications of *C. elegans* findings to mammalian system [100]. *C. elegans* expressing mutated version of human tau protein, which causes frontotemporal dementia with parkinsonism-17 (FTDP-17) in humans, ETX treatment ameliorated progressive loss in movement and reduced insoluble form of mutated tau protein levels. Similarly, in a model for lateral sclerosis (mutant TAR DNA-binding protein-43 in the GABAergic motor neurons; mTDP-43) ETX treatment reduced paralysis in a *daf-16*-dependent manner [113] (Table 1).

### Trimethadione– a succinimide based anticonvulsant

The succinimide anticonvulsant trimethadione (TRI) shares structural similarities with ETX and treatment of worms resulted in comparable phenotypes. Continuous drug treatment from larvae to death of worms or early treatment from young adult (L4 developmental stage) with TRI prolonged the animal’s lifespan (Fig. 3). Similar to ETX various healthspan parameters such as movement, pharyngeal pumping and early-stage egg laying have also showed improvement (Table 1). However, TRI was more potent compared to ETX to increase lifespan of loss-of-function mutants affecting sensory perception (*osm-3*,* tax-4*), neurotransmission (*unc-31*, *unc-64*, *aex-3*) and transmission of the insulin-like signal (*daf-2*, *daf-16*) suggesting the involvement of additional signalling pathways to exert the beneficial effect. On the other hand, TRI increased sensitivity to aldicarb, which paralyzes worms due to acetylcholine accumulation at the synapse, indicating increased excitability due to acetylcholine in the organism. Mutations or drugs that reduce synaptic transmission cause resistance to aldicarb [106]. Thus, TRI affects neuromuscular function as observed for ETX in the vulva muscles (Table 1). Given the similar responses of the worm to ETX and TRI, the effects of a combined treatment were investigated. Interestingly, co-treatment of ETX and TRI in wild-type worms at 2 mg/ml each showed an additive effect on lifespan extension. However, increased concentration of 4 mg/ml each resulted in a reduced improvement in lifespan compared to the single drug treatment [115]. This dose-dependent response illustrates that drug combinations may provide optimal beneficial effects at sub-maximal individual concentrations, conferring interesting insights for developing therapeutic interventions.

### Valproic acid - a fatty acid with anticonvulsant and anti-manic properties

Valproic acid (VA) is an anticonvulsant, but it does not share structural similarity with ETX and TRI (Table 1). It acts by inhibiting histone deacetylase enzymes (HDACs), which alter gene expression in humans [116]. VA at a concentration of 3 mM and 6 mM improved lifespan in wild-type worms exposed to the drug throughout life (Fig. 3). VA required *daf-16* to improve lifespan. VA treatment showed an additive effect on longevity with kanamycin, suggesting that the effectiveness of the drug is reduced when metabolised by the live bacteria (Table 1). VA treatment improved body movement of worms but did not stimulate egg laying like ETX or TRI. Therefore, VA can activate specific motor neurons that innervate different muscle groups. Surprisingly, combining VA treatment with longevity mutations including *daf-16*, *daf-2*, *eat-2* and *isp-1* resulted in a toxic response, which is strikingly different to ETX and TRI, but may indicate a different mode of action of these drugs (Table 1). The combination of VA (6 mM) with TRI (28 mM), both at the established optimal dosage, showed an additive effect on lifespan [115]. The epigenetic functionality seen in humans was not tested with respect to lifespan in worms, which would be an interesting intersection of biology with epigenetics and neuromodulation.

VA can be an important candidate for neuronal proteostasis regulation, as VA retained the morphological integrity of dopaminergic neurons expressing α-synuclein protein. The production of α-synuclein protein in dopaminergic neurons has been a model for Parkinson’s disorder in worms. VA treatment at a concentration of 3 mM showed protective capacity for dopaminergic neurons, which required the presence of *mek-2* and *mpk-1*, two genes of the ERK-MAPK pathway. This provides a good link between VA, the dopamine neurotransmission system and Parkinson’s disease [105].

VA treatment was studied in the lateral sclerosis model in *C. elegans* (mutant TAR DNA-binding protein-43 in the GABAergic motor neurons; mTDP-43) but, unlike ETX, it further increased the tendency to paralysis compared to no treatment in the mutant [113]. This shows that VA is not effective in improving healthspan in this model (Table 1).

## Antidepressant

### Mianserin – a tetracyclic antidepressant

A drug screen for extension of adult *C. elegans* lifespan revealed a class of drugs similar in structure to human serotonin antagonists. The candidate drug mianserin, which is an antidepressant prescribed to humans, increased the mean lifespan of worms in a dosage dependent manner (~ 34% at a 100 µM and ~ 23% at 50 µM) (Fig. 3; Table 1) [117]. Later studies confirmed that mianserin treatment specifically reduced mortality in young adults up to day 10, the post-reproductive period when many organismal functions begin to decline. A 10-day exposure to mianserin was sufficient to extend lifespan to the same extent as a lifetime exposure, suggesting that drug exposure at the onset of health decline is beneficial to the organism. Shorter exposures of 8 h and 24 h in young adulthood could extend lifespan, but not as much as longer exposures [118]. The drug administration timing suggested that the drug prevents the onset of aging based damages during the reproductive and early post-reproductive period. Mianserin treated worms showed delayed aldicarb-dependent paralysis (Table 1). This supports that it works presynaptically [118]. The lifespan extension by mianserin required the biosynthesis of the neuromodulator serotonin, as well as serotonin receptor SER-4, and the octopamine receptor SER-3. Pharmacological in vitro binding studies reported mianserin as an antagonist of both the serotonin receptor encoded by SER-1 and octopamine receptor SER-3 [114] (Table 1). Treatment with mianserin caused a significant decrease in the worm`s egg laying capacity, as serotonin is an egg laying stimulant for worms [117]. Mianserin regulates dietary restriction-induced *fmo-2* expression (*an* intestinal marker) by modulating serotonin signalling through SER-4 receptor in young *C. elegans*, demonstrating neurotransmitter-mediated control of dietary responses [119]. On the contrary, mianserin treatment for 10 days at a concentration of 50 µM increased body fat content in worms [120]. Increased appetite and body mass are also observed in humans when treated with serotonin antagonists [121]. It provides evidence for conserved serotonergic mechanisms between *C. elegans* and humans.

Development and aging cause a shift in the transcriptional expression levels called transcriptional drift [118]. Interestingly, mianserin exposure attenuated the transcriptional drift associated with aging in *C. elegans*. By day 10 of adulthood, wild-type worms exposed to mianserin had a slightly lower transcriptional drift variance than day 3 controls, suggesting an approximately 30% delay in age-related transcriptional changes [122]. Further evidence supported that mianserin improved worm`s stress resistance. Treated worms showed increased survival upon paraquat-induced oxidative stress. Mianserin required the serotonin receptor SER-5 for paraquat resilience. Oxidative stress resistance was dependent on active synaptic transmission. A later study argued that mianserin treatment attenuated the oxidative stress transcriptional signature in day 10 adults, consistent with preservation of redox homeostasis capacity rather than direct activation of the antioxidative stress response. Mianserin exposure also activated the intestine specific catabolic gene *ges-1*, which also was dependent on active neuronal function. Thus, the findings indicate that mianserin can influence cross-tissue signalling [118]. Mianserin promotes health through serotonin neurotransmission, but more potently through global transcriptional regulation.

## Antipsychotic drug

### Aripiprazole – piperazine and quinolone derivative-based antipsychotic

Aripiprazole (APZ) is an antipsychotic drug used in humans to treat schizophrenia, major depressive disorder, irritability related to autism, bipolar disorder and Tourette’s syndrome. APZ acts as a partial agonist for dopamine (dopamine D2 and D3) and serotonin receptors (serotonin 5-HT1a and 5-HT2a) [123, 124]. APZ binds to the receptors with high affinity reducing the activity of the receptors [125, 126]. *C. elegans* has a conserved dopamine system, which is similar to the mammalian nervous system. *C. elegans* DOP-1, DOP-4 (neuronal and non-neuronal expression) and DOP-2, DOP-3 (neuronal expression) are homologs of the human dopamine receptors, D1 and D2, respectively [127]. In *C. elegans*,* dop-2* mutants showed a reduction in lifespan whereas *dop-4* mutants promoted longevity. Thus, APZ in worms provides longevity mainly via inhibition of *dop-2* receptor [128]. The dopamine D2 receptor is a GPCR receptor, which uses $\:{G}_{\alpha\:o}\:$protein for activation of downstream signalling. The GPCR downstream signalling requirements were independently studied with mutants. APZ-induced effects did not depend on GOA-1 (immediate downstream protein) and cyclic AMP production for the lifespan extension. APZ-induced mechanisms downstream of *dop-4* receptor can require *egl-8* indicating that different GPCR downstream proteins might still play a role in the mechanism of action. Thus, APZ may exert allosteric function in modulating dopamine signalling as the drug requires dopamine molecules for its action [128] (Table 1).

APZ-induced longevity through other pathways depend on protein kinase C and D homologs, AMPK, and *daf-16*, the transcription factor for longevity improvements. APZ could not extend the lifespan of *eat-2* mutant, which causes caloric restriction due to inhibition of pharyngeal pumping. Thus, APZ does regulate metabolic and energetic processes during aging [128].

The dependence of APZ on dopamine and serotonin signalling was also examined by behavioral assays that relay on neurons enriched in dopamine and serotonin receptors. The gentle touch assay depends on anterior touch of the worm, which activates AVM and ALM neurons and posterior touch, which activates PVM and PLM neurons. In particular, AVM and PVM neurons depend on dopamine [129]. The effect of APZ on these two receptor groups was demonstrated as a decrease in the anterior soft-touch response to APZ at a concentration of 300 µM in young adults. APZ treatment reduced pharyngeal pumping in wild-type worms. APZ depended on *dop-1* and *dop-2* for decreasing gentle touch response and was independent of *ser-1* and *ser-2*. Serotonin dependence was tested by the pharyngeal pumping assay with single mutants and APZ required both receptors to show the reduction in phenotype [130]. These findings demonstrate the neuromodulatory potential of APZ, achieved via serotonin- and dopamine-mediated inhibition of motor neurons (Table 1).

APZ treatment has been studied in models of Machado-Joseph (MDJ) neurodegenerative disease in *C. elegans* [25, 26]. MDJ is a progressive neurological and motor decline disease, which is due to deterioration in the cerebellar and brainstem [131]. In *C. elegans*, expression of mutated human ataxin-3 protein with poly-glutamine repeats leads to aggregates and proteotoxicity in the neurons of worms. Worms show poor motility and the phenotype is used to test effectiveness of a drug to improve health. APZ at 10 µM showed no toxicity to the worms. APZ treatment showed a reduction of locomotion defects in the MDJ model and improved lifespan for the mutant worms after 4 days of exposure to the drug. However, APZ exposure did not decrease aggregate formation of the ATXN3 mutant protein. Co-treatment with chemical antagonists of *dop-2*,* ser-4* and inverse agonists of *ser-5*,* ser-7 and ser-1* showed a dose-dependent cancellation of APZ based locomotory health improvement. Thus, serotonin and dopamine receptors appear to be important for APZ-dependent healthspan improvement in the MDJ model [132]. These findings hint the protein homeostasis through neuromodulation in a disease condition (Table 1).

## Antihypertensive drug

### Reserpine – an alkaloid based antihypertension drug

Reserpine was one of the first oral antihypertensive drugs effective through depleting monoamine neurotransmitters [133–135]. Moreover, reserpine was used to treat schizophrenia and severe agitation because of its sedative and antipsychotic properties [135].

In *C. elegans*, reserpine (RSP) improved lifespan and healthspan determined by increased locomotion, pharyngeal pumping (after day 8 adulthood), thermo-tolerance at 35 °C and reduced brood size [136]. Nonetheless, RSP treatment during development showed some degree of toxicity in worms. Worms exposed to RSP at 60 µM concentration from the L1 larval stage on showed a decrease in survival and a marginal delay in the development time to reach adulthood. Treatment of worms from adult stage onwards showed improved locomotion up to day 5 of adulthood and removal of RSP before day 5 decreased the effect of improved locomotion in aging worms (Table 1). Pharmacogenetic interaction studies helped to elucidate neurotransmission-based mechanisms of RSP. RSP did not extend lifespan of *unc-104* mutant, a kinesin-1 involved in anterograde trans-synaptic signalling from the neuron body along the axon towards the synapse. The release of neurotransmitters depends on calcium-activated channels, such as SLO-1, a calcium-activated potassium channel. RSP did not extend the lifespan of *slo-1* mutants, highlighting the need for ion channels in synaptic transmission for its action [137]. In contrast, RSP did extend the lifespan of mutants of vesicular monoamine transporter, VMAT, encoded by *cat-1* gene and mutants of dopamine and serotonin synthesis, *bas-1* and *cat-2*, which indicates that monoamine transport and neurotransmitter synthesis were not essential for RSP action. RSP provided resistance to aldicarb in acute and chronic exposure assays in wild-type worms indicating that the drug acts presynaptically. RSP is an antihypertensive drug, which is dependent on the neurotransmitter acetylcholine. Lifespan extension by RSP was abolished in hypomorphic mutation of acetylcholine synthesis (*cha-1*) and acetylcholine packing into synaptic vesicles (*unc-17*). Unlike most other neuromodulating drugs discussed in this review, RSP improves healthspan independent of the stress resilience and longevity pathways activated through *daf*-*16* and in the *eat-2* mutant, which causes dietary restriction (Table 1) [137].

RSP treatment in models of neurodegeneration expressing amyloid beta (Aβ_1−42_) peptide showed improvement of healthspan and lifespan. Paralysis of these worms was reduced by 50% when animals were treated from L4 stage and by 43% when treatment started from day 2 of adulthood [138]. RSP did not reduce the transcript levels and aggregates levels of Aβ_1−42_ peptide [137, 138]. Similarly, RSP treatment reduced paralysis of worms in the model for lateral sclerosis [expression of mutant TAR DNA-binding protein-43 (mTDP-43) in the GABAergic motor neurons] in *C. elegans* but the drug action appeared to be independent of *skn-1*,* daf-16*,* sir-2.1 and hsf-1* [113]. Altogether, a molecular mechanism by which RSP improves movement in these models remains to be shown.

## NSAID

### Acetylsalicylic acid – Calorie restriction mimetic and salicylate NSAID

Acetylsalicylic acid (ASA), commonly known as Aspirin, regulates pain, fever, and inflammation in humans by irreversibly inhibiting cyclooxygenases (COX) within the prostaglandin system. In contrast, *C. elegans* lacks cyclooxygenase homologs but synthesizes high levels of F-series prostaglandins through an alternative, yet unidentified, COX-independent pathway [139, 140]. Thus, a mechanism of action remains to be described in *C. elegans*. Nevertheless, ASA extends the lifespan of *C. elegans* and improves the health of worms [102, 141, 142]. Compared to other NSAIDs (ibuprofen, acetaminophen and indomethacin), ASA prolonged life with the highest median lifespan. Healthspan markers such as coordinated movement, head sway and pharyngeal pump frequency were improved from young to old time points in adulthood with ASA treatment [95] (Table 1). This finding hints at the activation of motor neurons during organismal aging. Pharmacogenetic interactions measured over the lifespan revealed a dependence on *daf-2* and *daf-16* (insulin-like pathway), *clk-1* and *isp-1* (reduced mitochondrial respiration), as well as *eat-2* and *aak-2* (dietary restriction and metabolism) (Table 1). ASA is a calorie restriction mimetic in *C. elegans*. The increased AMP: ATP levels in worms upon ASA treatment confirms the dietary restriction mediated effect [142]. Caloric restriction is one of the most conserved interventions providing longevity [143–145]. At the transcriptomic level, ASA treatment resulted in the up-regulation of genes involved in metabolic, developmental and reproductive pathways. Metabolically, ASA treatment increased transcript levels of genes involved in lipid hydrolysis and fatty acid β-oxidation. Lipid metabolic modulation was mediated via *daf-12.* ASA treatment from young adulthood requires germline signalling of GLP-1 to extend lifespan. However, it reduced the number of germline stem cells in the hermaphrodites [141]. This might indicate that it requires somatic and reproductive tissue crosstalk. ASA treated worms showed higher thermo-tolerance but on the contrary, levels of *hsp-16.2* gene at day 5 and day 12 of adulthood were decreased. ASA caused a significant reduction of reactive oxygen species (ROS) in the organism measured on day 5 and day 12 of adulthood alongside higher expression levels of antioxidant genes (*sod-1*,* sod-2*,* sod-3)* (Table 1). Unlike other NSAIDs, increased oxidative stress response upon ASA treatment was independent of *skn-1* transcription factor giving ASA a unique edge as a drug option [95]. Thus, ASA could provide neuroprotective effects through stress resilience.

Responses upon ASA treatment were also linked to autophagy in *C. elegans*. Autophagy can be monitored in *C. elegans* by utilizing a GFP reporter strain of LGG-1, an autophagy cargo tethering protein, forming puncta upon autophagy activation [146]. Alongside, a known autophagy substrate, SQST-1, was decreased in the adult head/pharynx upon exposure to ASA. Exposure to ASA increased autophagic puncta formation even when autophagy was decreased by inhibitor bafilomycin A1. Knockdown of essential autophagy genes (*bec-1* and *atg-7*) by RNA interference abolished the increase in LGG-1 puncta upon ASA exposure. ASA did not increase autophagic puncta formation when *cbp-1* (the *C. elegans* ortholog of EP300 acetyltransferase) was knocked down. This demonstrates that ASA acts through the known autophagy genes. Moreover, ASA also promotes selective autophagy-mediated degradation of mitochondria [147] (Table 1).

ASA treatment at a concentration of 500 µM was shown to improve chemosensation in young adult worms expressing various neurodegenerative model proteins (tau, Aβ_1−42_, TDP-43). ASA treatment slowed the paralysis of worms expressing Aβ_1−42_ in muscles and worms showed a decrease in amyloid aggregates. Similar results were obtained for a polyglutamine (PolyQ) model expressing PolyQ40 fused to yellow fluorescent protein (YFP). ASA treatment reduced the number of aggregates in worms on day 1 compared to salicylic acid, a structural analog. From an aging perspective, ASA reduced the intensity of amyloid aggregates on a 2D gel from day 1 to day 7, but did not change total protein levels [102]. Since neurodegenerative proteins were expressed in the worm muscles, no conclusion can be drawn about the neuroprotective effect of ASA, but it highlights the drug`s potency for proteostasis protection (Table 1).

The neurological impact of ASA in humans arises from its anti-inflammatory function via COX-1 and COX-2 inhibition through acetylation [148, 149]. Notably, ASA preferentially inhibits COX-1, which are implicated in neurodegeneration and neuroinflammation, potentially making it a candidate for modifying disease progression in neuropsychiatric disorders. ASA has been explored for its therapeutic potential in depression, schizophrenia, mood disorders, and Alzheimer’s disease, showing some promise in improving neuropsychiatric symptoms, though risks of side effect remain to be considered [reviewed in [150].

### Celecoxib – a benzene derivate based NSAID

Celecoxib (CLX) is a non-steroidal anti-inflammatory drug and specifically blocks cyclooxygenases − 2 (COX-2) in humans [151]. CLX in worms showed an improvement in lifespan at a concentration of 10 µM from egg hatching with a 30% increase and at 2 µM for adult-only exposure with a 10–15% increase in lifespan (Table 1). However, potential toxic effects were reported at concentrations higher than 200 µM [152]. A structural analog, OSU-03012 (OSU), which lacks the COX-2 inhibitory function, was used to dissect the mechanism. OSU improved lifespan at 0.5 µM for lifelong exposure and adult stages with ~ 15–25% increase compared to control. This indicates that the main structural component of the drug is critical for the lifespan improvement in *C. elegans* [153].

CLX was found to act via insulin-like pathway and *daf-16*. However, CLX and OSU dependent lifespan extension was independent of *eat-2* (calorie restriction), *pdk-1* (IIS downstream kinase) and *cyc-1* (mitochondrial respiration). CLX and OSU reduced the phosphorylation of *pdk-1* target *sgk-1*, implying an inhibitory effect on the IIS pathway [153]. CLX improved the lifespan of mutant *ins-7*, an insulin-like peptide with potential agonistic functional with *daf-2*. Interestingly, CLX acts via *daf-16* and upregulates *ins-7* over 24 h to 14 days. On contrary, it is known that *ins-7* is downregulated when *daf-16* is active and CLX has extended lifespan without *ins-7*. CLX showed differential effects of the two classes of *daf-16* targets. As a class II target gene, CLX has a unique response on *ins-7* via upregulation, as this gene is downregulated upon *daf-*16 activation. The *ins-7* is also regulated by another transcription factor (*pqm-1*) in absence of *daf-16*. CLX increased *ins-7* in the worm intestine upon *daf-16* knockdown, which was verified via INS-7::GFP localization. This highlights an interesting perspective on IIS pathway and pharmacological treatment [154]. CLX has the potential to improve health through metabolic rewiring and longevity activation through the modulation of the IIS pathway (Table 1). However, the mitochondrial function of CLX can still be explored during aging.

OSU reduced the number of aggregate foci in PolyQ35::YFP worm strains, suggesting potential proteostasis protection. OSU and CLX provided health benefits in a *C. elegans* tumor model. The tumor model is set up in the germline due to excessive proliferation of cells in the *gld-1* mutant (Table 1). The compound/drug reduced the number of germline-based tumors in young adults [153]. CLX, like ASA has the potential for neuroprotective function due to its ability to maintain protein homeostasis.

Similar to ASA, CLX has been investigated for its potential to decelerate disease progression in neuropsychiatric disorders like major-depressive disorder, obsessive-compulsive disorder and other mood disorders. As an adjunct to psychiatric mediation, CLX shows potential for its use in anti-inflammation-based therapeutics for neuropsychiatric health [155–157].

## Limitations of *C. elegans* for translational neurobiology

The model *C. elegans* offers a great platform for generating hypotheses on how drugs might improve human health. However, successful translational research requires multimodal validation, the establishment of dosing correlations, the study of multiple healthspan parameters, and the identification of translatable biomarkers. Fundamental biological differences between *C. elegans* and human neurobiology need to be considered as well. *C. elegans* has a completely mapped connectome for both hermaphrodites and males, offering detailed insights into its nervous system architecture [23, 158–160]. However, in contrast to humans, neurons in *C. elegans* communicate without myelinated axons and neurons have fewer numbers of synaptic connections [159]. Glial cells are comparatively sparse and mainly localized to sensory structures near the head [161]. Although neuropeptides in *C. elegans* have pleiotropic effects on tissue physiology [162] with some translational relevance to human neuromodulation [163], the neurotransmitter repertoire in *C. elegans* is limited compared to humans lacking key molecules such as histamine, epinephrine, and norepinephrine. Furthermore, *C. elegans* relies on multifunctional sensory neurons critical for its survival responses instead of specialized sensory organs as found in humans [36, 71, 164]. The absence of complex brain regions and emotional behaviour further distinguishes the worms from humans. Moreover, the hermaphrodite–male system does not adequately capture sex-based differences in neural function because it does not fully cover the chromosomal and hormonal complexity of dimorphic sexes in humans [55, 165, 166]. Aging trajectories differ markedly between *C. elegans* and humans. Both species exhibit age-related declines in neuronal activity, as shown by calcium signaling studies in *C. elegans* and brain imaging in humans [46, 167]. However, humans maintain several aspects of cognitive function through compensatory neural mechanisms during aging [168], whereas *C. elegans* display a rapid post-reproductive decline without evidence of analogous compensatory circuitry [9, 169].

From a pharmacological perspective, the absence of a blood-brain barrier in *C. elegans* allows direct access of compounds to neurons, although this limits its translational relevance to humans, where the blood-brain barrier is a major determinant of central nervous system drug efficacy. Conversely, the nematode’s protective cuticle and lack of a circulatory system restrict drug absorption and distribution making it difficult to correlate administered dose with internal exposure or target engagement. This limits dose-response assessments and comparisons with human pharmacokinetics [170, 171]. Behavioral assays are widely used to evaluate the effects of neuromodulatory drugs but face several limitations. Most rely primarily on measures of locomotor activity, which are significantly impaired in aged worms, thereby complicating the interpretation of sensory and cognitive functions. In addition, *C. elegans* behavior is highly sensitive to environmental conditions, genetic background (particularly in motor-defective mutants), and experimental variability. These factors collectively reduce reproducibility and confound the assessment of neurobehavioral drug effects and age-related functional decline [172]. As reported by Lucanic et al., only a small fraction of longevity-promoting compounds produced consistent positive effects across three independent laboratories. This finding demonstrates the need for standardized experimental protocols that account for variables such as *C. elegans* strain genetic background, bacterial food source, culture density, humidity, temperature fluctuations, and handling procedures [172].

## Conclusions and future directions

**Fig. 4:**
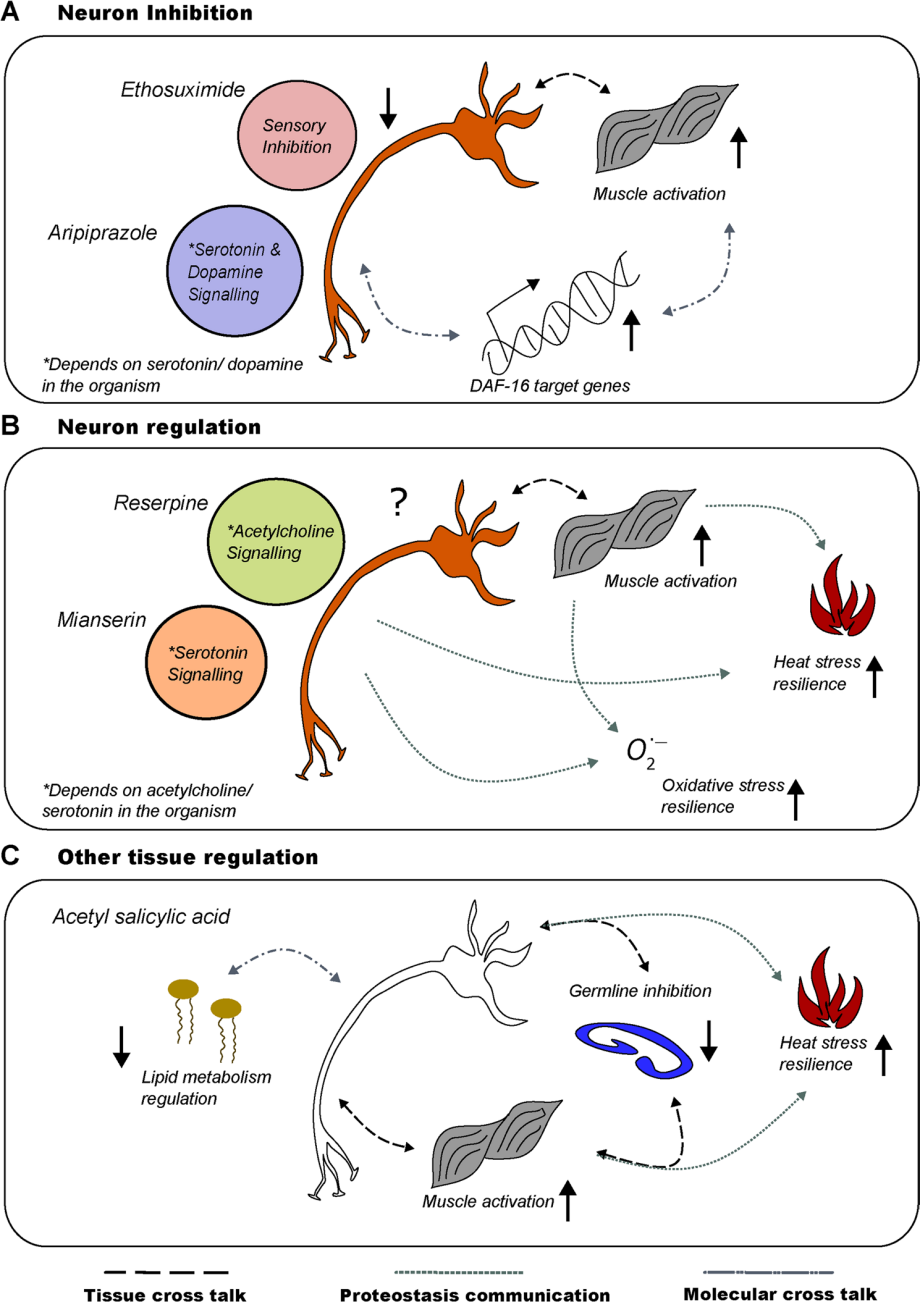
Mechanism of different drugs for healthspan improvement. **A** Neuronal inhibition-based health improvements. Ethosuximide and aripiprazole downregulate neuronal function and neurotransmitter-related pathways. **B** Neuron regulation-mediated health improvements. Reserpine and mianserin require neurotransmitter pathways to provide health benefits. **C** Regulation of other tissues. Acetylsalicylic acid does not directly affect neurons but regulates muscle and germline function. Black arrows denote activation (up) and inactivation (down). The black question mark denotes no specific direction of neuronal modulation. The three types of dashed lines depict the different types of cellular, molecular, and tissue cross talk that are activated by drug treatment

Despite the limitations, *C. elegans* is a useful model because of the strong conservation of fundamental biological mechanisms [23, 109, 158–160, 173]. These conserved mechanisms provide a foundation for translating discoveries from invertebrate models into therapeutic prospects for humans. The neuronal network is critical for the health of the organism due to its functional connectivity with other tissues. The drugs discussed in this review demonstrate their function as neuromodulating drugs through their geroprotective and neuroprotective mechanisms. Anticonvulsants exhibit broad phenotypes by modulating neuro-muscular activity and chemosensory neurons, while antipsychotics affect the serotonin and dopamine systems by inactivating the touch neurons and pharyngeal neurons (Fig. 4A). Antidepressants depend on serotonin neurotransmission, but there is evidence of neuronal modulation through serotonin, acetylcholine and dietary restriction. Antihypertensive drugs modulate acetylcholine and general neurotransmission, proving to be broad neuromodulators (Fig. 4B). In addition, nonsteroidal anti-inflammatory drugs (NSAIDs), used primarily to treat inflammation, also benefit *C. elegans* at the molecular level by inhibiting the insulin-like signalling (IIS) pathway, which indirectly affects neuromodulators and consequently behaviour and survival (Fig. 4C). All these drugs provide health and longevity benefits through conserved mechanism of modulating the IIS pathway, transcriptional regulation, metabolic rewiring, mitochondrial functionality, autophagy, activation of stress response along with unfolded protein response to combat disruption of cellular homeostasis. Additionally, *C. elegans* is a powerful screening model with translational applications due to its ability to reveal complex drug actions and its use in understanding neuromodulation through neurodegenerative protein models. Current observations suggest that no single intervention can completely reverse all age-related changes; while some neurons may regain health, other cellular and molecular mechanisms may only partially return to a healthier state. Thus, drug-repurposing research must adopt parallel approaches that take into account tissue crosstalk and bioavailability, drug-binding targets within the *C. elegans* proteome and interactions with the gut microbiome, since the bacteria on which *C. elegans* are cultured in the laboratory can metabolise many drugs. Fundamental research questions to be addressed include the identification of molecular mechanisms underlying neuron-to-tissue signaling during aging and drug intervention; whether combinatorial drug treatments targeting multiple neuromodulatory pathways are more effective in improving neuronal health and longevity than single-drug approaches; the long-term effects of neuromodulatory drugs on neuronal plasticity and functional recovery during aging; and the optimal timing and dosage parameters for neuromodulation and longevity. Recently artificial intelligence (AI)-driven approaches improved the rational design of polypharmacological compounds targeting multiple biogenic amine receptors and achieving enhanced efficacy in extending lifespan compared to single-target drugs, suggesting polypharmacology may be important for developing exceptional neuromodulators and geroprotectors [174].

## Data Availability

The data presented in Figure 3 were curated from DrugAge database based on build 5 (29.11.2024). Data for the depicted drugs are based on the following publications: Ethosuximide [106, 107], Trimethadione [106, 175], Valproic acid [115, 176], Mianserin [96, 117], Reserpine [136, 137, 177], Aspirin/Acetylsalicylic Acid [141, 142, 178], Celecoxib [153], Ibuprofen[49], Fluvoxamine [179], Octoclothepin maleate [96], Nortriptyline hydrochloride [96], Dihydroergocristine methanesulfonate [96], Methylergonovine maleate [96], Metergoline [96], Dihydroergotamine methanesulfonate [96], Amoxapine [96], Pergolide methanesulfonate [96], and Guanfacine hydrochloride [96].

## Abbreviations

MAPK: Mitogen-Activated Protein Kinase
mTOR: Mechanistic/mammalian Target of Rapamycin
TGF-β: Transforming Growth Factor beta
GABA: Gamma-Aminobutyric Acid
ANCL: Autosomal-dominant adult-onset Neuronal Ceroid Lipofuscinosis
mTDP-43: Mutant TAR DNA-binding protein-43
FTDP-17: Frontotemporal Dementia with Parkinsonism-17
MDJ: Machado-Joseph neurodegenerative disease
NSAID: Non-Steroidal Anti-Inflammatory Drug
ETX: Ethosuximide
TRI: Trimethadione
VA: Valproic Acid
APZ: Aripiprazole
RSP: Reserpine
ASA: Acetyl Salicylic Acid
CLX: Celecoxib
OSU: OSU-03012
